# Quality and Preparation of Pediatric Patients for Kidney Transplantation: Experience at Prince Sultan Military Medical City

**DOI:** 10.7759/cureus.98951

**Published:** 2025-12-11

**Authors:** Mugahid Elhag Elamin, Bashair Alabbasi, Ghada Alzahrany, Azizah Alanazi, Abdulmonem Alghamdi, Shouq Naif Aloufi, Majed Aloufi

**Affiliations:** 1 Department of Pediatric Nephrology, Prince Sultan Military Medical City, Riyadh, SAU; 2 Faculty of Medicine, Taibah University, Medina, SAU

**Keywords:** multidisciplinary approach, pediatric kidney transplantation, pediatric transplant outcomes, psmmc, quality of care

## Abstract

Background and objective

Kidney transplantation is the preferred treatment for children with end-stage kidney disease (ESKD), offering superior survival, quality of life, and growth outcomes compared with dialysis. Achieving successful outcomes requires thorough preparation and strict adherence to standardized protocols. This study aimed to report the quality measures and standardized preparation protocol for pediatric kidney transplantation at Prince Sultan Military Medical City (PSMMC), Riyadh, Saudi Arabia.

Methods

This manuscript describes the multidisciplinary protocol for pediatric transplant candidates and patients undergoing evaluation, including assessment of dialysis modality, pretransplant optimization, donor evaluation, immunological workup, perioperative management, and posttransplant care. Both hemodialysis and peritoneal dialysis are used as bridging modalities until transplantation. Kidney transplant types include deceased-donor kidney transplant, living-donor kidney transplant, living-related donor transplant, living-unrelated donor transplant, and preemptive kidney transplantation. At PSMMC, approximately 10-15 pediatric kidney transplants are performed annually.

Results

PSMMC implements a comprehensive, multidisciplinary approach to ensure quality and safety. This includes structured medical, nutritional, psychosocial, and immunological preparation; regular multidisciplinary team meetings; infection prophylaxis according to strict guidelines; and a clearly defined immunosuppression management plan. The availability of both living and deceased donor programs improves access and equity of care.

Conclusions

The pediatric kidney transplant program at PSMMC aligns with international quality assurance standards and national healthcare policy. Thorough pretransplant evaluation, close multidisciplinary collaboration, and weekly team meetings facilitate follow-up and early detection of complications. Benefits include improved nutrition and growth, consistent immunosuppression, robust infection prophylaxis, and long-term postoperative management. The inclusion of both living and deceased donor programs provides families with greater choice, shorter wait times, and a family-centered approach to psychosocial care, enhancing compliance and long-term graft survival. A formalized transition from pediatric to adult nephrology care, initiated at age 14, ensures continuity of care. This structured, phased protocol fosters autonomy in adolescents, supporting sustained transplant success. Collectively, these strategies enhance graft longevity, patient well-being, and a smooth transition from childhood to adulthood. Ongoing advancements in clinical protocols and transition models further establish PSMMC as a leading center for pediatric kidney transplantation in Saudi Arabia, recognized for exemplary patient-centered care and long-term outcomes.

## Introduction

Kidney transplantation is a well-established and sophisticated treatment for pediatric patients with end-stage kidney disease (ESKD), offering superior long-term survival and quality of life compared with dialysis. However, both hemodialysis (HD) and peritoneal dialysis (PD) are associated with significant challenges. Beyond their physical impacts, dialysis modalities can impose substantial psychological burdens, particularly for children [1-4]. In contrast, transplantation restores normal physiological function more comprehensively than merely addressing physiologic needs. Children who undergo transplantation experience more favorable physical and psychological recovery, supporting improved growth and developmental outcomes compared with dialysis [3].

Successful pediatric kidney transplantation requires more than surgical expertise. Optimal outcomes depend on thorough pretransplant evaluation, meticulous perioperative management, and standardized posttransplant care. Comprehensive preparation encompasses medical optimization, immunological workup, nutritional support, vaccinations, infection prophylaxis, psychosocial readiness, and careful donor selection. Achieving sustained graft function and favorable long-term survival necessitates the integration of these elements within a multidisciplinary framework [1,2].

Prince Sultan Military Medical City (PSMMC) in Riyadh is a leading tertiary center with an established pediatric nephrology and kidney transplant program. The center performs approximately 10-15 pediatric kidney transplants annually, including living-donor, living-related, living-unrelated, deceased-donor, and preemptive transplantation. A prior study from PSMMC (2009-2017) reported effective posttransplant follow-up and growth outcomes in pediatric recipients [4].

This paper describes the preparation protocols and quality benchmarks that guide pediatric kidney transplantation at PSMMC. We highlight our collaborative, multidisciplinary approach and the integration of both living and deceased donor programs, all designed to optimize clinical outcomes, graft longevity, and the overall well-being of pediatric patients.

## Materials and methods

The preparation protocol for pediatric kidney transplantation at PSMMC, Riyadh, Saudi Arabia, is summarized in this narrative. The recommendations follow the guidelines of the Saudi Center for Organ Transplantation (SCOT) and international standards, drawing from Kidney Disease: Improving Global Outcomes (KDIGO) [5,6]. On average, the PSMMC pediatric kidney transplant program performs 10-15 transplants annually, utilizing both living-related and deceased donor organs. This approach ensures adherence to evidence-based practices and aligns with contemporary pediatric transplant strategies reported worldwide [7]. The study protocol was reviewed and approved by the Institutional Review Board of PSMMC (IRB approval no. E-2675, dated September 16, 2025).

Inclusion and exclusion criteria

All pediatric patients under 14 years of age with ESKD who were evaluated for kidney transplantation at PSMMC were considered for this study. The inclusion and exclusion criteria are summarized in Table 1.

**Table 1:** Inclusion and exclusion criteria for pediatric kidney transplantation candidates at PSMMC ESKD, end-stage kidney disease; HD, hemodialysis; PD, peritoneal dialysis; PKT, preemptive kidney transplantation; PSMMC, Prince Sultan Military Medical City

Category	Criteria
Inclusion criteria	Pediatric patients aged <14 years with ESKD evaluated for kidney transplantation at PSMMC
	Candidates undergoing comprehensive pretransplant assessment, regardless of prior kidney replacement therapy modality, including HD, PD, and PKT
Exclusion criteria	Patients with incomplete medical records or missing essential pretransplant data
	Candidates undergoing multi-organ transplantation
	Patients or families who declined transplantation
	Children with contraindications to transplantation, including active malignancy, uncontrolled or severe infection, and a significant risk of nonadherence to medical therapy

PSMMC performs kidney transplantation from multiple donor categories: deceased-donor kidney transplant, living-related donor transplant, living-unrelated donor transplant, and preemptive kidney transplantation (PKT).

Preparation and quality measures

Patient Selection and Evaluation

Children with ESKD are assessed as transplant candidates. Contraindications, such as active malignancy, severe or uncontrolled infection, or significant risk of nonadherence, are carefully excluded. A comprehensive evaluation includes a detailed medical history, systemic examination, cardiac assessment, infectious disease screening, and consultations with dental, ENT, and kidney transplant surgery specialists [8].

Immunological, Laboratory, and Radiological Assessment

All candidates undergo human leukocyte antigen typing, crossmatching, and panel-reactive antibody testing [9]. Baseline investigations include hematologic, kidney, liver, and metabolic profiles to identify and correct abnormalities prior to transplantation. A detailed thrombotic workup and infectious risk stratification are performed, focusing on cytomegalovirus (CMV) and Epstein-Barr virus status, along with other infection risks as indicated. Radiological assessment, specifically abdominal vascular mapping via CT angiography/CT venography, is conducted to detect vascular malformations or chronic thrombosis.

Nutritional and Growth Optimization

Nutritional status is assessed through anthropometric measurements. Interventions include dietary supplementation, correction of anemia and vitamin D deficiency, and management of metabolic bone disease. Endocrine and dietary support is provided to optimize growth and development before transplantation [10].

Donor Evaluation

Living-related donors are preferred when available, while deceased donors are allocated through SCOT if a living donor is not possible [5]. Donor evaluation includes kidney imaging, GFR determination, and a comprehensive psychosocial and ethical assessment to ensure donor safety and eligibility.

Pretransplant Dialysis Optimization

For children on HD or PD, dialysis adequacy is ensured with attention to volume control, electrolyte stability, and infection prevention. PKT is considered in selected patients to avoid dialysis exposure.

Perioperative Preparation

Patients are admitted 24-48 hours before transplantation. Preoperative measures include repeat cross-matching, administration of prophylactic antibiotics, and optimization of comorbidities.

Immunosuppression Protocol

Induction therapy: Basiliximab is used routinely, while anti-thymocyte globulin is reserved for patients at high immunological risk.

Maintenance therapy: The standard regimen includes tacrolimus, mycophenolate mofetil, and tapering corticosteroids.

The immunosuppressive regimen protocol for pediatric kidney transplantation at PSMMC follows standardized induction and maintenance time points, as summarized in Table 2.

**Table 2:** Immunosuppression drug dosage protocol before and after kidney transplantation ^*^ Mycophenolate mofetil: Begin one day prior to transplantation at a dose of 600 mg/m² per administration, given twice daily. Once the target tacrolimus level is achieved, reduce the dose to 300 mg/m² per day, administered in two divided doses. ^**^ Tacrolimus: Start once the allograft demonstrates adequate function, indicated by a serum creatinine level below 250 µmol/L or a reduction of more than 50% from baseline. Adjust the dosage according to the target therapeutic level. This table outlines the medication protocol for all pediatric kidney transplant recipients. ATG, anti-thymocyte globulin; PSMMC, Prince Sultan Military Medical City Source: Elamin et al. (2024) [4]

Medication	Dose	Frequency
ATG (mg/kg) for deceased kidney transplant, Days 0-3	4.5	Cumulative dose
Basiliximab (mg/kg) for living kidney transplant, Days 0 and 4	<35 kg: 10 mg ≥35 kg: 20 mg	Two doses
Mycophenolate mofetil^*^ (mg/m²)	600 initially, then 300	Every 12 hours
Tacrolimus^**^ (mg/kg/day), start Day 1	0.3	Every 12 hours
Prednisolone (mg/kg/day)		
Day 0	200 mg/m²	Given in recovery
Day 1	2	Every six hours
Day 2	1.75	Every six hours
Day 3	1.5	Every six hours
Day 4-6	1.25	Every 12 hours
Day 7-9	1	Every 12 hours
Day 10-12	0.75	Every 12 hours
Day 13-14	0.5	Every 12 hours
Week 3	0.45	Once daily
Week 4	0.4	Once daily
Week 5	0.3	Once daily
Week 6-7	0.25	Once daily
Week 8-9	0.2	Once daily
Week 10-12	0.15	Once daily
Week 13-16	0.1	Once daily
Week >16	0.1 mg/kg/dose	Once daily

Posttransplant monitoring

The pediatric nephrology team receives the patient directly from the operating room and remains with the child in the recovery area until transfer to the pediatric ICU (PICU). The immediate focus is on fluid management, with meticulous estimation of replacement fluids, as pediatric patients vary in their native kidney urine output. Underestimating fluid replacement may cause dehydration, leading to impaired graft perfusion and function. Serial Doppler ultrasound assessments begin intraoperatively and are repeated upon arrival to the PICU to evaluate graft perfusion and vascular integrity. Ongoing monitoring includes strict fluid balance, recording of urine output, daily weight measurements, and serial laboratory evaluations, including creatinine and electrolytes. Prophylaxis includes trimethoprim-sulfamethoxazole (for *Pneumocystis jirovecii *pneumonia), valganciclovir (for CMV), and antifungal coverage when indicated. Rejection surveillance involves clinical examination, laboratory assessments, Doppler imaging, and biopsy when clinically indicated [11].

Quality and Safety Framework

The multidisciplinary transplant team is actively involved in patient care. Weekly team meetings, including consultants, residents, transplant coordinators, and nursing staff, review patient files, follow-up data, and update the active waiting list. Regular case audits and morbidity/mortality reviews are conducted, and patient and family education on adherence is emphasized. Outcomes are benchmarked against SCOT and international registries.

Family-Centered Care

Pediatric kidney transplantation impacts not only the patient but also the entire family, particularly in cases of living-related donation where both donor and recipient undergo surgery simultaneously. The same nephrology team that receives the patient from the operating room also meets with the parents and family in the recovery and PICU setting.

Transition From Pediatric to Adult Care

Coordinated transition to adult nephrology is crucial for maintaining long-term transplant success. Transition is not a single event but a systematic, staged process that empowers patients to take charge of their health care. At PSMMC, transition begins at age 14, per institutional policy.

Early preparation: Children are prepared from late childhood and early adolescence, gradually introduced to management skills appropriate for their age, including knowledge of medications, warning signs, and follow-up procedures.

Multidisciplinary approach: Pediatric nephrologists, nurses, psychologists, social workers, and transplant coordinators collaborate to provide comprehensive medical and psychosocial education. Parents are encouraged to gradually give patients greater independence in self-managing their health.

Formal handover: Patients are formally transferred to adult nephrology at 14 years of age. Handover meetings and shared documentation ensure continuity of care and prevent interruptions.

Timing of transition: The optimal age for transfer varies by institution, is patient-dependent, and is influenced by the regional health care system. Evidence suggests that the best outcomes are achieved through a stepwise, individualized transition plan rather than relying solely on the child’s age. The International Society of Nephrology and the International Pediatric Nephrology Association “Standards of Care” recommend transition to adult care at 16-18 years, with flexibility to account for the patient’s maturity, health status, and local policies [12].

Table 3 summarizes key considerations supporting the timing of transition.

**Table 3:** Outlines the key steps and main points involved in transitioning from pediatric to adult care

Timing of transition	Age span	Pros/advantages	Cons/considerations
Early transition	≤14 years	Aligns with institutional policy; safe and effective with preparation and structured handover	Requires robust programs; risk of poor adherence if preparation is inadequate
Middle transition	15-17 years	Most commonly used internationally; allows more time to develop self-management skills	May still require close support; needs strong coordination between pediatric and adult teams
Late transition	18-21 years	Greater maturity and independence; potentially beneficial for self-management	Risk of compromised outcomes if transition is not well planned; requires careful preparation
Best practice	Individualized	Gradual, flexible, maturity-based transfer model	Chronological age alone should not dictate timing; requires a multidisciplinary approach

Results/outcomes

Barriers among early adolescents include noncompliance with medication, difficulty for some families in accepting transition at a young age, and differences in practices between pediatric and adult services. Despite these challenges, the systematic transition protocol at PSMMC facilitated a smoother transfer of care, reduced stress for families, and ensured stable and appropriate graft outcomes.

International perspective

Globally, the age for transition to adult services is typically higher, commonly between 16 and 21 years [13,14], allowing more time for adolescent development and fostering patient autonomy. The PSMMC policy of transferring patients at 14 years aligns with local health care regulations and hospital policy. With advanced preparation and structured planning, care can continue safely and effectively.

## Results

The pediatric kidney transplantation program at PSMMC is founded on a holistic, multidisciplinary approach, dedicated to delivering high-quality care, ensuring patient safety, and promoting long-term graft survival. The program integrates comprehensive medical, nutritional, psychosocial, and immunological assessments, complemented by weekly team meetings that allow in-depth candidate review. Proactive measures, including robust infection prevention protocols and a standardized immunosuppression regimen, are central to optimizing each patient’s outcomes.

The inclusion of both living and deceased donor programs ensures equitable access to transplantation. Comprehensive psychosocial support is provided throughout all phases of care, including family counseling, stress management, structured feedback, and reminders for medication adherence and follow-up. This patient- and family-centered approach is a hallmark of the PSMMC program, contributing to superior pediatric kidney transplant outcomes, as summarized in Table 4.

**Table 4:** Key components of the PSMMC pediatric kidney transplant multidisciplinary program PSMMC, Prince Sultan Military Medical City

Domain	Key components
Medical	Standardized pre- and posttransplant evaluation; infection prophylaxis according to institutional guidelines
Nutritional	Comprehensive nutritional assessment and individualized dietary planning
Psychosocial	Family counseling, stress and anxiety management, structured feedback to enhance engagement
Immunological	Human leukocyte antigen typing, crossmatching, and individualized immunosuppression planning
Coordination	Weekly multidisciplinary team meetings for case discussion and follow-up
Posttransplant support	Medication reminders, adherence reinforcement, and continuous monitoring
Donor program	Integrated living and deceased donor programs to promote equitable access

## Discussion

The pediatric kidney transplantation program at PSMMC represents a quality-based model that integrates international best practices with national healthcare needs. Performing an average of 10 to 15 pediatric kidney transplants annually, the center constitutes a significant proportion of Saudi pediatric transplant activity and serves as a referral hub for complex cases. Inclusion of both living and deceased donor programs, in collaboration with the SCOT, expands transplant opportunities and reduces wait times for children with ESKD [5].

A key strength of the PSMMC program is its multidisciplinary approach. Weekly transplant team meetings, including consultants, residents, coordinators, and transplant nurses, review patient cases, follow-up data, and updates to the waiting list. This team-based strategy enables seamless care continuity, early detection of complications, and patient-centered management. Similar multidisciplinary frameworks in international centers are associated with improved graft survival and patient outcomes [15].

Pretransplant preparation is another hallmark of the program. Rigorous patient selection and exclusion criteria, coupled with thorough immunological evaluation, minimize perioperative risks. Nutritional and growth optimization addresses common comorbidities in children with ESKD, such as anemia, metabolic bone disease, and malnutrition. Optimizing growth before transplantation is particularly critical, as better pretransplant nutritional status consistently correlates with improved posttransplant growth and development [16].

Immunosuppression at PSMMC aligns with international standards, with basiliximab induction and tacrolimus-based triple therapy as the backbone of maintenance regimens [17]. This is complemented by comprehensive infection prophylaxis covering *P. jirovecii*, cytomegalovirus, and antifungals. Serial monitoring, including laboratory evaluations, Doppler imaging, and, when necessary, needle biopsy, facilitates early detection and management of rejection, consistent with global recommendations such as those from KDIGO [18]. Routine audits, morbidity and mortality reviews, and benchmarking against SCOT and international registries further strengthen quality and safety [5], providing accountability and fostering continuous improvement.

Family-centered care is another distinguishing feature of the PSMMC program. Recognizing the dual surgical involvement in living-donor transplantation, the nephrology team provides real-time psychosocial support to families, reducing anxiety, enhancing trust, and promoting adherence to long-term therapy. Collectively, these strategies enhance surgical success, graft function, psychosocial well-being, and long-term graft survival. By combining international best practices with tailored interventions, the PSMMC pediatric kidney transplant program serves as a sustainable model for quality improvement in resource-integrated healthcare systems [19].

Strengths and limitations

This study represents one of the earliest Saudi series on pediatric kidney transplantation and provides detailed insight into pretransplant evaluation and preparation. Use of standardized protocols and close patient monitoring strengthens the reliability of the findings. The structured preparation and transition protocols may serve as a model for other centers. Limitations include its single-center design, which may limit generalizability, and the small cohort size, reflecting the relative rarity of pediatric kidney transplantation. Additionally, long-term outcomes beyond the peri-transplant period were not extensively assessed, highlighting the need for multicenter studies and extended follow-up.

## Conclusions

The pediatric kidney transplant program at PSMMC adheres to international quality standards and national healthcare policy. Comprehensive pretransplant evaluation, multidisciplinary collaboration, and weekly team meetings ensure effective follow-up and early detection of complications. Improved nutrition, consistent immunosuppression, robust infection prophylaxis, and long-term postoperative management contribute to optimized outcomes. The combination of living and deceased donor programs offers families greater choice, shorter waitlist times, and a family-centered approach to psychosocial care, enhancing compliance and long-term graft survival.

A formalized transition from pediatric to adult nephrology care, initiated at age 14 per institutional policy, ensures continuity of care. This structured, phased protocol promotes adolescent autonomy, supporting sustained transplant success. Together, these strategies enhance graft longevity and patient well-being and facilitate a smooth transition from childhood to adulthood. Ongoing refinement of clinical protocols and transition models further establishes PSMMC as a leading center for pediatric kidney transplantation in Saudi Arabia, recognized for exemplary patient-centered care and long-term transplant outcomes.
